# Heme oxygenase-2 gene deletion attenuates oxidative stress in neurons exposed to extracellular hemin

**DOI:** 10.1186/1471-2202-5-34

**Published:** 2004-09-17

**Authors:** Raymond F Regan, Jing Chen, Luna Benvenisti-Zarom

**Affiliations:** 1 Department of Emergency Medicine, Thomas Jefferson University, 1020 Sansom Street, 239 Thompson Building, Philadelphia, PA 19107, USA

## Abstract

**Background:**

Hemin, the oxidized form of heme, accumulates in intracranial hematomas and is a potent oxidant. Growing evidence suggests that it contributes to delayed injury to surrounding tissue, and that this process is affected by the heme oxygenase enzymes. In a prior study, heme oxygenase-2 gene deletion increased the vulnerability of cultured cortical astrocytes to hemin. The present study tested the effect of HO-2 gene deletion on protein oxidation, reactive oxygen species formation, and cell viability after mixed cortical neuron/astrocyte cultures were incubated with neurotoxic concentrations of hemin.

**Results:**

Continuous exposure of wild-type cultures to 1–10 μM hemin for 14 h produced concentration-dependent neuronal death, as detected by both LDH release and fluorescence intensity after propidium iodide staining, with an EC_50 _of 1–2 μM; astrocytes were not injured by these low hemin concentrations. Cell death was consistently reduced by at least 60% in knockout cultures. Exposure to hemin for 4 hours, a time point that preceded cell lysis, increased protein oxidation in wild-type cultures, as detected by staining of immunoblots for protein carbonyl groups. At 10 μM hemin, carbonylation was increased 2.3-fold compared with control sister cultures subjected to medium exchanges only; this effect was reduced by about two-thirds in knockout cultures. Cellular reactive oxygen species, detected by fluorescence intensity after dihydrorhodamine 123 (DHR) staining, was markedly increased by hemin in wild-type cultures and was localized to neuronal cell bodies and processes. In contrast, DHR fluorescence intensity in knockout cultures did not differ from that of sham-washed controls. Neuronal death in wild-type cultures was almost completely prevented by the lipid-soluble iron chelator phenanthroline; deferoxamine had a weaker but significant effect.

**Conclusions:**

These results suggest that HO-2 gene deletion protects neurons in mixed neuron-astrocyte cultures from heme-mediated oxidative injury. Selective inhibition of neuronal HO-2 may have a beneficial effect after CNS hemorrhage.

## Background

Hemin is a potent oxidant that accumulates in intracranial hematomas and may contribute to neural cell injury [1,2]. It is also the preferred substrate for heme oxygenase-2, the constitutively-expressed isoform that accounts for most CNS heme oxygenase (HO) under normal conditions [3]. In pathologic states, HO frequently has an antioxidant effect, putatively due to the protection provided by increased cellular bilirubin, decreased heme, and up-regulation of other antioxidants [4-7]. However, in models that are relevant to CNS hemorrhage, HO inhibitors have surprisingly been found to be protective [8-10].

All HO inhibitors that are currently available have numerous non-specific actions that may complicate the interpretation of experimental results, including inhibition of nitric oxide synthase and guanyl cyclase, and modification of voltage-gated calcium currents [11-14]. Some may also have a direct antioxidant effect that is unrelated to HO inhibition [15]. In order to investigate HO-2 in heme-mediated injury more specifically, we have cultured neurons and astrocytes derived from HO-2 knockout mice and genetically-similar wild type controls. In recent studies, we observed that astrocytes derived from mutant mice were more vulnerable to hemin [16]. Conversely, HO-2 gene deletion decreased the vulnerability of neurons to hemoglobin [17]. Neither wild type nor knockout astrocytes were injured by hemoglobin at the micromolar concentrations that are feasible in vitro. HO-2 gene deletion per se did not result in a compensatory increase in HO-1 in these cultures, and produced minimal or no change in other cellular antioxidants [16,17].

The disparate effects of HO-2 gene deletion on hemin toxicity to astrocytes and hemoglobin toxicity to neurons may reflect the inability of neurons to tolerate the products of heme metabolism, i.e. iron, carbon monoxide, and bilirubin. Alternatively, it may reflect the different oxidant properties of hemin and hemoglobin. Although the oxidant effect of hemoglobin may be due in part to hemin release to membrane lipids [18], other mechanisms may also contribute. Extracellular hemoglobin undergoes autoxidation, which produces superoxide [19]. In addition to being an oxidant, superoxide reacts with globin amino acids in a complex fashion to generate a variety of reactive species, including thiyl radicals, hydroxyl radicals, and hydrogen peroxide [20,21]. It is also noteworthy that hemoglobin is highly water soluble while hemin is quite lipophilic; their accumulation in separate cellular compartments may lead to a different pattern of site-specific oxidative damage [22,23].

The present study was designed to test the effect of HO-2 gene deletion on the oxidative neuronal injury produced by extracellular hemin. We specifically tested the hypothesis that targeted deletion of the HO-2 gene attenuated oxidative cell injury in a primary cell culture model of hemin toxicity.

## Results

### Effect of HO-2 gene deletion on hemin neurotoxicity

**Figure 1:**
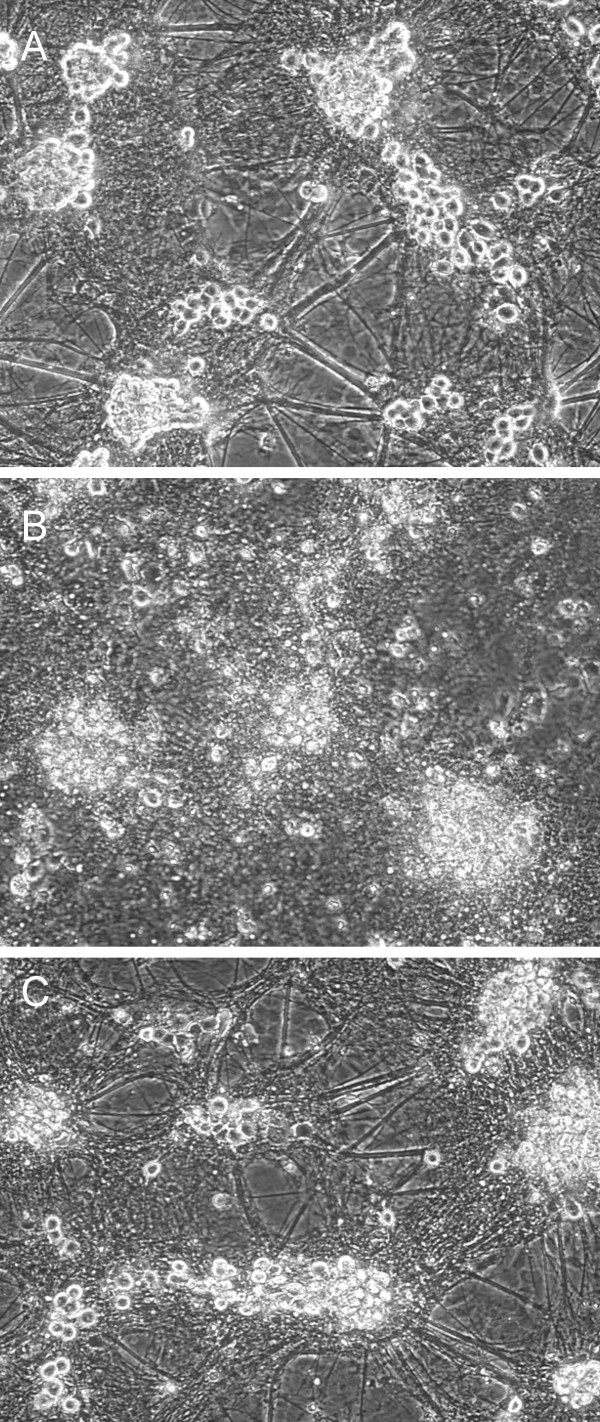
Morphologic changes in wild type and knockout cultures exposed to hemin. Phase contrast photomicrographs of cultures exposed for 14 h to: A) Experimental medium (MEM10) only; neurons rest on a monolayer of astrocytes, and aggregate in groups which send out an array of processes; B) hemin 3 μM, wild type culture; most neurons and processes have degenerated; C) hemin 3 μM, knockout culture; neurons with intact processes persist.

**Figure 2:**
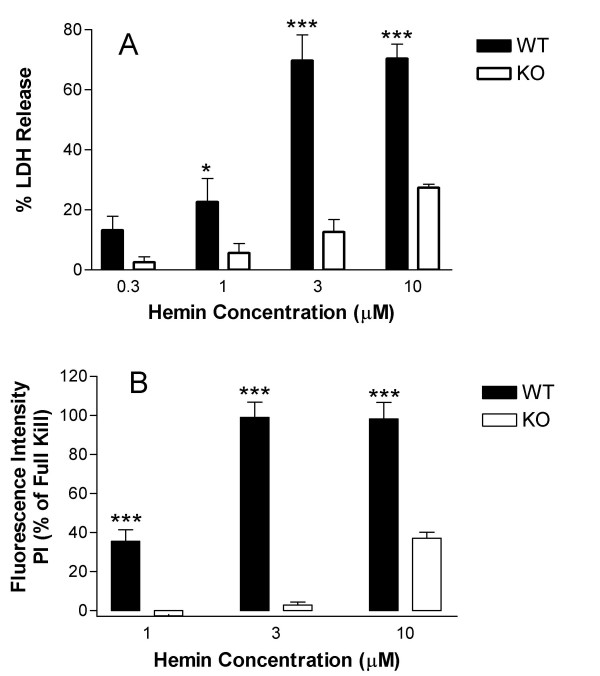
Heme oxygenase-2 gene deletion attenuates the neurotoxic effect of hemin. Cultures were treated with indicated concentrations of hemin for 14 h. Injury was assessed by A) LDH activity in the medium, which is scaled to that in sister cultures treated with 300 μM NMDA for 40 h (= 100), which releases essentially all neuronal LDH; B) fluorescence intensity of cultures stained with propidium iodide, again scaled to that in sister cultures treated with NMDA. *P < 0.05, ***P < 0.001 v. knockout cultures treated with same concentration of hemin, Bonferroni multiple comparisons test.

In preliminary experiments, we observed that overnight (14 h) exposure to low micromolar concentrations of hemin consistently produced morphologic evidence of neuronal injury in wild-type cultures (Fig. 1). This time interval was therefore used for cytotoxicity studies. Consistent with prior observations in pure astrocyte cultures [24], no morphologic evidence of injury was observed in the astrocyte monolayer at hemin concentrations up to 10 μM. In order to specifically assess neuronal injury in this study, the concentrations used were limited to this range. In wild-type cultures, cell injury as quantified by LDH release was observed at 1 μM hemin and then increased exponentially, to release of 69.7 ± 8.6% of neuronal LDH at 3 μM (Fig 2A). The calculated EC_50 _was 1.85 μM. LDH release was significantly reduced in knockout cultures subjected to the same treatment. At 3 μM hemin, only 12.6 ± 4.1% of LDH had been released at this time point. Control experiments demonstrated that these low hemin concentrations do not interfere with the LDH assay.

Cell death was also quantified by analysis of fluorescence intensity after staining cultures with propidium iodide. Using this method, widespread neuronal death was also observed at 3–10 μM hemin in wild type cultures, and the calculated EC_50 _was 1.05 μM. Propidium staining of nuclei was significantly reduced in cultures prepared from HO-2 knockout mice (Fig. 2B). The maximal fluorescence was produced by exposure to 10 μM hemin, which was 37.0 ± 3.2% of that observed in control sister cultures treated with NMDA to kill all neurons.

### Effect of HO-2 gene deletion on markers of cell oxidation

**Figure 3:**
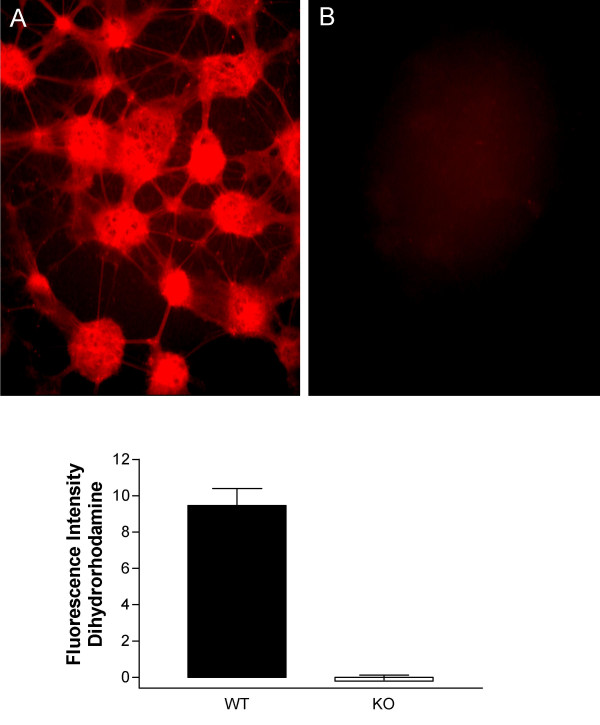
Heme oxygenase-2 gene deletion reduces production of cellular reactive oxygen species, assessed with dihydrorhodamine 123. Cultures were treated with 3 μM hemin for 4 h. Hemin was then washed out, and cultures were treated with 20 μM dihydrorhodamine 123 for 15 min and imaged. A) Wild type culture; fluorescence is localized to neuronal cell bodies and processes; B) Knockout culture; fluorescence is markedly diminished. Fluorescence intensity is quantified in arbitrary units.

In order to assess reactive oxygen species formation after hemin exposure, cultures were stained with 20 μM dihydrorhodamine 123 after 4 hour hemin exposure. This time interval was used because it preceded cell lysis, and therefore allowed cell retention of the reduced fluorophore. A marked increase in fluorescence was observed in cultures prepared from wild type mice (Fig. 3). This signal was concentrated in neuronal cell bodies and processes. In contrast, fluorescence in cultures prepared from HO-2 knockout mice was minimal, and did not exceed that observed in cultures subjected to medium exchanges only.

**Figure 4:**
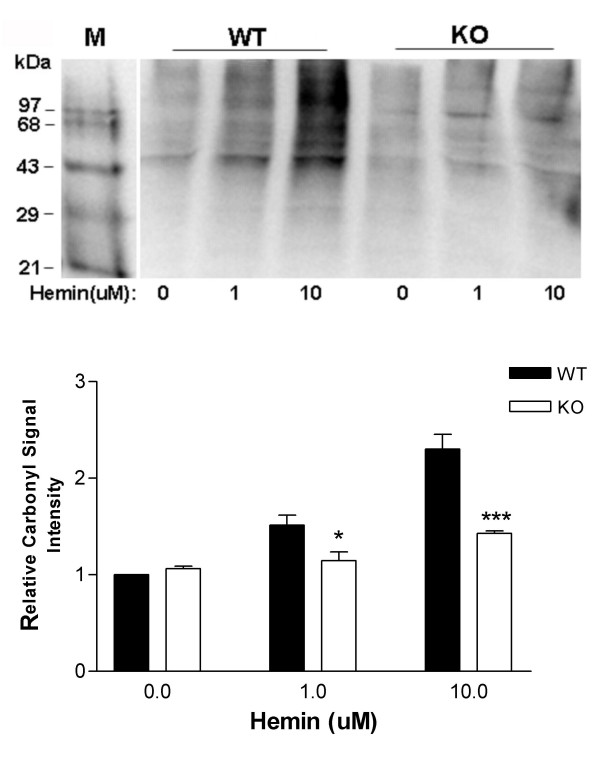
Heme oxygenase-2 gene deletion reduces protein oxidation in hemin-treated cultures. Top: Representative immunoblot of protein lysates from wild type (WT) or HO-2 knockout (KO) neuron/astrocyte cultures treated for 4 h with indicated hemin concentrations, stained with anti-DNP antibody to detect derivatized carbonyl groups. M: molecular weight standard with attached DNP residues. Bottom: The mean protein carbonyl signal intensity (± SEM, n = 5/condition) was normalized to that in wild-type cultures exposed to culture medium only (= 1.0) *P < 0.05; ***P < 0.001 versus signal in wild type cultures exposed to the same hemin concentration, Bonferroni multiple comparisons test.

In order to further investigate the effect of HO-2 gene deletion on oxidative stress produced by hemin, cells were harvested after 4 hour hemin exposure. Protein carbonyl groups (i.e. aldehydes and ketones), which are markers of oxidation, were then derivatized and detected with a dinitrophenylhydrazone antibody. Increased immunoreactivity was apparent in lysates of wild type cultures treated with hemin (Figure 4). A prominent band was present at approximately 44 kDa, along with a higher molecular weight smear. At 10 μM hemin, the carbonyl signal intensity in wild type cultures was 2.3-fold higher than in cultures exposed to culture medium only, compared with only 1.3-fold higher in knockout cultures.

### Hemin neurotoxicity is attenuated by iron chelators

**Figure 5:**
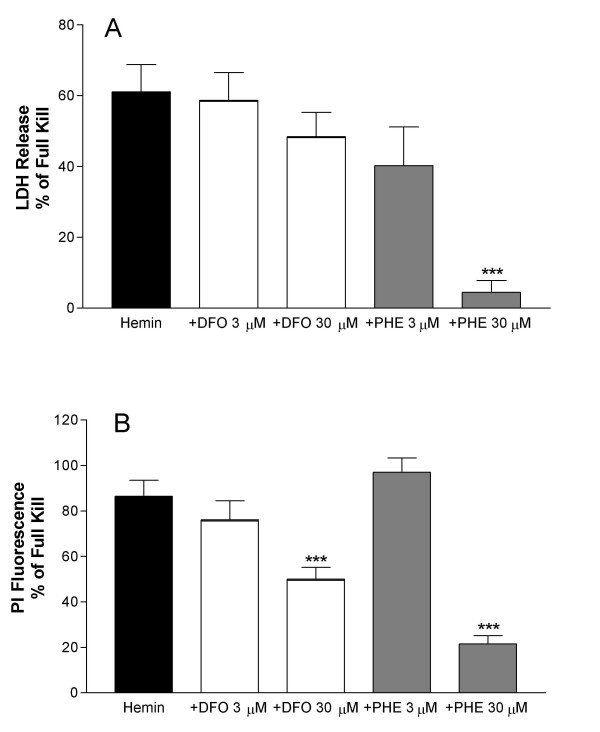
Effect of iron chelators on hemin toxicity. Wild-type cultures were treated with 3 μM hemin for 14 h, alone or with indicated concentrations of deferoxamine (DFO) or phenanthroline (PHE). Injury was assessed by A) LDH activity in the culture medium, which is scaled to that in sister cultures treated with 300 μM NMDA for 40 h (= 100), which releases essentially all neuronal LDH; B) fluorescence intensity of cultures stained with propidium iodide, again scaled to that in sister cultures treated with NMDA. ***P < 0.001 v. cultures treated with hemin only, Bonferroni multiple comparisons test.

Based on our prior observations in astrocytes and neuroblastoma cells [24,25], we hypothesized that the toxic product produced by hemin breakdown in primary murine neurons was iron. In order to test this hypothesis, the effect of iron chelators on hemin neurotoxicity in wild type cultures was assessed. Most cell death, as detected by both LDH release and PI staining, was prevented by concomitant treatment with phenanthroline, a lipid-soluble iron chelator (Fig. 5). Deferoxamine, which is water soluble, was less potent; its effect when applied at a concentration tenfold greater than that of hemin reached statistical significance only when injury was assessed by PI staining.

## Discussion

In prior experiments, we demonstrated that targeted deletion of the HO-2 gene in primary neuron/astrocyte cultures did not alter expression of HO-1, and had little or no effect on other cellular antioxidants [17]. This simplified system therefore permits investigation of HO-2 without the confounding compensatory effects that have been observed in whole animal models [26,27]. We have previously reported that HO-2 gene deletion increased the vulnerability of astrocytes to hemin, the preferred substrate of HO, in cultures containing only astrocytes. The present study targeted neurons in mixed neuron/astrocyte cultures by using hemin concentrations that did not injure astrocytes [24]. In this model, the opposite was observed. HO-2 deletion attenuated hemin-induced ROS formation and reduced levels of oxidized proteins. Consistent with the oxidative nature of hemin toxicity, neuronal death was reduced in knockout cultures.

Although hemin is a highly reactive pro-oxidant, its breakdown as catalyzed by the heme oxygenases generates biologically active and potentially toxic products. Prior in vitro studies suggest that neurons are particularly vulnerable to these, i.e. iron, carbon monoxide, and bilirubin [28-30]. The present results suggest that when neurons are provided with an excess of HO substrate, the toxicity of breakdown products outweighs any benefit provided by hemin removal. The protective effect of iron chelators suggests that this phenomenon is at least partly due to iron neurotoxicity. Inorganic iron is toxic to cultured cortical neurons, with an EC_50 _of approximately 10 μM [17]. The lower EC_50 _for hemin is not surprising, given its lipophilicity and accumulation in cell membranes [31]. The latter phenomenon likely accounts for the greater efficacy of phenanthroline, which unlike deferoxamine is lipophilic. Deferoxamine is quite effective against hemoglobin neurotoxicity in this culture system [32], suggesting that hemoglobin releases its iron either in the medium or in an aqueous cellular compartment.

The present results are consistent with observations that heme oxygenase inhibitors are protective in models of CNS hemorrhage [2,8,9], in contrast to the beneficial effect of HO in ischemia [33]. In a recent study, Koeppen et al. [2] observed that repeated administration of tin mesoporphyrin protected thalamic neurons from the delayed degeneration that occurred in tissue adjacent to injected autologous blood. Similarly, Huang et al. [9] observed that tin protoporphyrin attenuated edema formation after stereotactic hemoglobin injection into the rat striatum. It is noteworthy that the number of astrocytes per neuron is significantly higher in the human brain than in rodents [34]. The deleterious effect of HO inhibition on heme mediated injury to astrocytes may therefore be less prominent in animal models than in clinical intracerebral hemorrhage [35].

The disparate effect of HO on neurons and astrocytes exposed to extracellular hemin suggests that it may be somewhat difficult to target it effectively after CNS hemorrhage. All currently available HO inhibitors inhibit both HO-2, which is predominantly neuronal in vivo [36], and HO-1, which is induced mainly in glial cells [37]. The protection that these non-selective agents provide to neurons may be negated by their deleterious effect on astrocytes. Further investigation is needed for the development of strategies that would permit the selective inhibition or down-regulation of HO-2 in neurons.

## Conclusions

Targeted deletion of the heme oxygenase-2 gene mitigates oxidative stress in cultured neurons exposed to hemin, and is cytoprotective. Selective inhibition of neuronal heme oxygenase may have a beneficial effect after CNS hemorrhage.

## Methods

### Cell cultures

The HO-2 knockout mice which were used in this study are descended from mutants produced by Poss et al. [38], and have a C57BL/6 X 129/Sv genetic background. All mice were obtained from our local breeding colony, and were provided with food and water ad libitum and a 12 hour light/dark cycle. All breeding mice were the offspring of heterozygotes. Genotype was determined by polymerase chain reaction (PCR) using genomic DNA isolated from tail clippings; primers were previously published [17].

Cortical cell cultures were prepared from fetal mice at gestational age 15–17 d as previously described [39]. Under a dissecting microscope, cortices were dissected free from other brain tissue, minced with forceps, and incubated in medium containing 0.075%-acetylated trypsin at 37°C for one hour. Tissue was then collected by low speed centrifugation, and was dissociated by trituration through a flamed Pasteur pipette in plating medium containing Eagle's minimal essential medium (MEM), 5% fetal bovine serum (Hyclone, Logan, UT), 5% heat inactivated equine serum (Hyclone), glutamine (2 mM), and glucose (23 mM). The cell suspension was diluted with additional plating medium, and cells were plated on confluent astrocyte cultures in 24-well plates (Primaria, Falcon) at a density of 3 hemispheres/plate. Cultures were incubated at 37°C in a humidified atmosphere containing 5% CO2/95% air. Two-thirds of the culture medium was replaced at days 4 and 8 in vitro with MEM containing 10% equine serum, 2 mM glutamine, and 23 mM glucose. After ten days in vitro this feeding procedure was performed daily.

### Hemin exposure

Hemin was freshly prepared as a 1 mM stock solution and was diluted to the desired concentration with minimal essential medium containing 10 mM glucose (MEM10). Experiments were conducted at 12–16 days in vitro. Serum was washed out of cultures with MEM10 (> 1000X dilution) prior to addition of hemin. Cultures were incubated at 37°C in a 5% CO2 atmosphere for the entire exposure interval.

### Detection of reactive oxygen species

ROS formation was quantified by staining with dihydrorhodamine 123 (DHR, Molecular Probes, Eugene, OR), which is a cell-permeable, non-fluorescent compound that is oxidized by cellular peroxides to fluorescent rhodamine [40]. Fluorescence intensity is directly proportional to cellular oxidative stress. In order to prevent oxidation of DHR by hemin in the medium, cultures were washed free of hemin prior to DHR addition. After incubation with 20 μM DHR in MEM10 for 15 min, the medium was replaced, and cultures were imaged using a Nikon inverted microscope with epifluorescence attachment. Images were captured immediately after illumination (25 msec exposure). Photomicrographs of random 100X fields were analyzed with IPLab image analysis software (Scanalytics, Inc., Fairfax, VA). The low fluorescence in control cultures exposed to experimental medium only was subtracted from mean values to define the signal associated with hemin exposure.

### Detection of protein oxidation

Protein oxidation was assessed using the Oxyblot™ kit (Chemicon, Inc., Temecula, CA). At the end of the hemin exposure interval, culture medium was aspirated, and cells were washed and then harvested in 100 μl of lysis buffer containing 210 mM mannitol, 70 mM sucrose, 5 mM HEPES, 1 mM EDTA, and 0.1% sodium dodecyl sulfate. Carbonyl groups were derivatized to 2, 4-dintrophenylhydrazone (DNP-hydrazone) by reaction with 2, 4-dinitrophenylhydrazine, following the manufacturer's instructions. Proteins were then separated on a 12% polyacrylamide gel and were transferred onto a polyvinylidene difluoride Imobilon-P transfer membrane filter (Millipore, Billerica, MA) using a semidry transfer apparatus (Bio-Rad, Hercules, CA). Carbonylated proteins were detected with rabbit anti-DNP (1:150) followed by goat anti-rabbit IgG (1:300). Immunoreactive proteins were visualized using Super Signal West Femto Reagent (Pierce Biotechnology, Rockford, IL) and Kodak ImageStation 400.

### Quantification of cell death

After examination of cultures using phase contrast microscopy, cell death was quantified by measurement of LDH activity in the culture medium, as previously described [41]. To facilitate comparisons, values were scaled to the mean value in sister cultures exposed to NMDA 300 μM for 40 h. This treatment releases essentially all neuronal LDH in this system without injuring astrocytes [42]. Since the low micromolar concentrations of hemin that were used in this study do not injure cultured cortical astrocytes [24], the contribution of astrocyte LDH to the total signal is negligible.

Cell death was also quantified by staining with propidium iodide (13 μg/ml for 15 min). When viewed with a rhodamine filter, the nuclei of cells with disrupted membranes stain red, while cells with intact membranes exclude propidium. Random 100X fields were captured with a Nikon Diaphot epifluorescence microscope and were analyzed with IPLab image analysis software. As with LDH data, fluorescence intensity was scaled to that in sister cultures treated with NMDA 300 μM for 40 h, which kills all neurons. Propidium iodide fluorescence was not observed in cells that had an astrocyte phenotype after treatment with NMDA or hemin at the concentrations used in this study.

## Abbreviations

DHR: dihydrorhodamine; DNP: dinitrophenylhydrazone; HO: heme oxygenase; LDH: lactate dehydrogenase; MEM10: minimal essential medium containing 10 mM glucose; NMDA: N-methyl-D-aspartate; PI: propidium iodide; ROS: reactive oxygen species.

## Authors' contributions

RFR designed the study, collected and analyzed data, and wrote the manuscript. JC also participated in data collection and analysis, and edited the manuscript. LBZ participated in genotyping and edited the manuscript. All authors reviewed and approved the final manuscript.
